# Advanced Presentation of GEMIN5 Neurodevelopmental Disorder in an Elderly Female

**DOI:** 10.7759/cureus.115273

**Published:** 2026-08-27

**Authors:** Min Thant Thaw, Hnin Thida Nwe, Kyaw Thura, Kyawt Kay Khine Wynn, Ashraghi Mohammad, Helen Grote

**Affiliations:** 1 General Internal Medicine, West Middlesex University Hospital, London, GBR; 2 Neurology, West Middlesex University Hospital, London, GBR; 3 Neurology, Institute of Neurosciences, NHS Greater Glasgow and Clyde, Glasgow, GBR

**Keywords:** cerebellar atrophy, gemin5, gem nuclear organelle-associated protein 5, nedcam, neurodevelopmental disorders, neurology

## Abstract

GEMIN5, an RNA-binding protein, recognizes small nuclear RNAs and delivers them to the survival motor neuron complex for assembly of small nuclear ribonucleoproteins, which is vital for the maintenance of motor neurons. Loss-of-function mutations in the GEMIN5 gene are associated with neurodevelopmental disorders. Biallelic GEMIN5 variants cause motor-predominant developmental delay and cerebellar atrophy. Clinically, GEMIN5 variants have been associated with neurodevelopmental disorder with cerebellar atrophy and motor dysfunction syndrome, which is characterized by developmental and cognitive delay, ataxia, motor dysfunction, hypotonia in infancy, and cerebellar atrophy. We report the case of a 79-year-old woman who presented with progressive ataxia, spastic paraparesis, contractures, bulbar dysfunction, and dementia on a background of learning difficulties. Two pathogenic variants in the GEMIN5 gene were identified by whole-genome sequencing following MRI findings of striking frontotemporal atrophy in addition to cerebellar atrophy.

## Introduction

Neurodevelopmental disorder with cerebellar atrophy and motor dysfunction (NEDCAM), caused by biallelic GEMIN5 variants, is characterized by global developmental delay with prominent motor abnormalities such as hypotonia in infancy, gait ataxia, and spasticity [1]. Affected individuals can have cognitive impairment. The severity of symptoms such as developmental delay and cognitive impairment can be variable between individuals. Brain imaging commonly shows cerebellar atrophy. It is an exceptionally rare genetic disorder. It was only in 2021 that the GEMIN5 gene was identified as the cause of the phenotype. While it was first reported in 2021, a total of 48 cases have been published in the literature at the time of writing [2]. Both truncating and missense biallelic variants have been reported to cause the disorder [3]. GEMIN5 is the largest subunit of the survival motor neuron (SMN) complex and functions both in small nuclear ribonucleoprotein assembly and in the independent control of mRNA translation [4].

## Case presentation

A 79-year-old Caucasian woman presented with progressive ataxia, spastic paraparesis, contractures, dysarthria, dysphagia, and dementia on a background of learning difficulties. She was born at term and achieved her early motor milestones. She had mild learning difficulties and was able to read and write; however, she was not able to perform higher cognitive functions such as mathematics. She attended a special school. Motor incoordination had been present since childhood, but it was attributed at the time to her learning difficulties and to being left-handed. She had a history of epilepsy during childhood and early adult life. These symptoms were static throughout her life.

**Figure 1:**
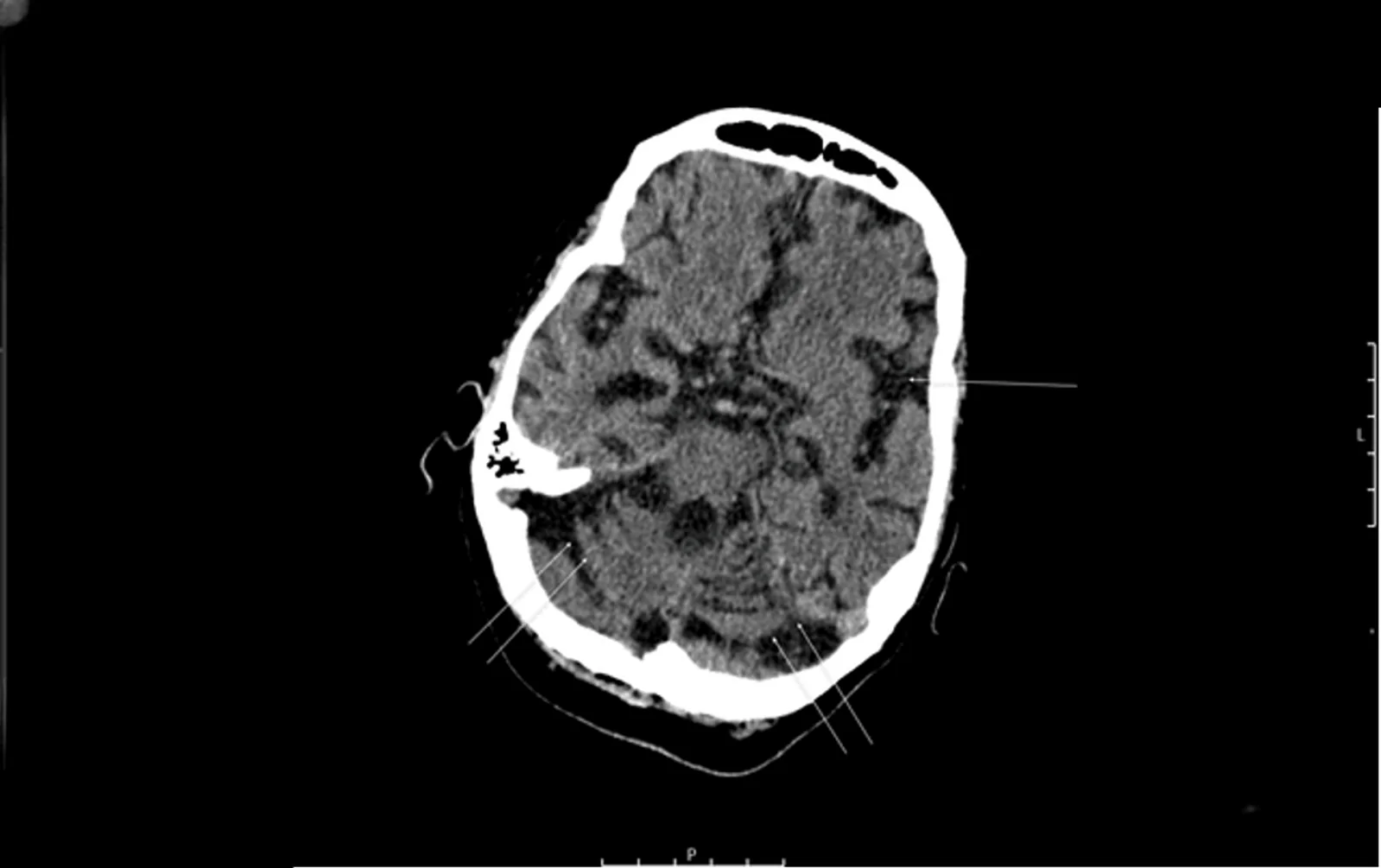
CT of the head performed at the age of 72. White arrows show moderate neuroparenchymal atrophy, more prominent in the cerebellum.

She married at the age of 23, and her husband managed most domestic and financial affairs. Her functional dependence increased progressively. At the age of 62, following her husband’s death, the extent of support she required with activities of daily living became apparent. At the time, she was noted to have ataxia and dysarthria, which prompted referral to the neurology clinic. On assessment at the clinic, she had a wide-based gait and used a three-wheeled walker. She had scanning dysarthria and oculomotor dyspraxia without nystagmus. Limb tone was normal. There was proximal hip-flexion weakness, although distal limb strength was preserved. MRI of the head performed at the age of 62 did not identify a cause for her ataxia. Autoimmune investigations, including antiphospholipid syndrome antibodies, antinuclear antibodies, antineutrophil cytoplasmic antibodies, and angiotensin-converting enzyme, were negative. Her memory decline was first noticed in her early to mid-60s, and she was referred to the memory clinic for assessment. CT of the head at the age of 72 demonstrated moderate neuroparenchymal atrophy, which was more prominent in the cerebellum (Figure 1).

From the age of 74, she had become increasingly stiff and immobile with spastic dysarthria. At the age of 76, she developed contractures and spastic paraparesis. The hand contractures were worse on the right than on the left. She developed tremors affecting both arms and legs and required the use of a wheelchair. At the age of 76, she was assessed in clinic by a neurogenetic specialist. She attended the appointment in a wheelchair. She was anarthric and required a Level 5 diet for dysphagia.

Examination demonstrated marked spasticity in both upper and lower limbs. Contractures were present, with fixed flexion at the elbow, wrist, and fingers of the right arm, and at the elbow and fingers of the left arm. Sensation to pinprick was intact in all four limbs. Reflexes were present in the upper limbs and at the knees but absent at the ankles. Plantar responses were upgoing bilaterally.

The patient was one of a sibship of four. Her brother (78) and sister (74) were well, with no learning difficulties or signs of a neurodegenerative condition. Another brother (72) possibly has learning difficulties and some difficulties understanding social norms around interacting with others. She has three children, one of whom has learning difficulties but without additional features to suggest a neurodegenerative disorder. Her other two children are both well and do not have learning difficulties. Her parents were not known to have any neurodegenerative conditions. Her father died of cardiac disease, and her mother died of lung cancer. There was no history of consanguinity. Genetic testing was not undertaken in the affected family members during the patient’s lifetime; during preparation of this case report, we signposted the family to their general practitioner for referral to clinical genetics should they wish to pursue cascade testing.

**Figure 2:**
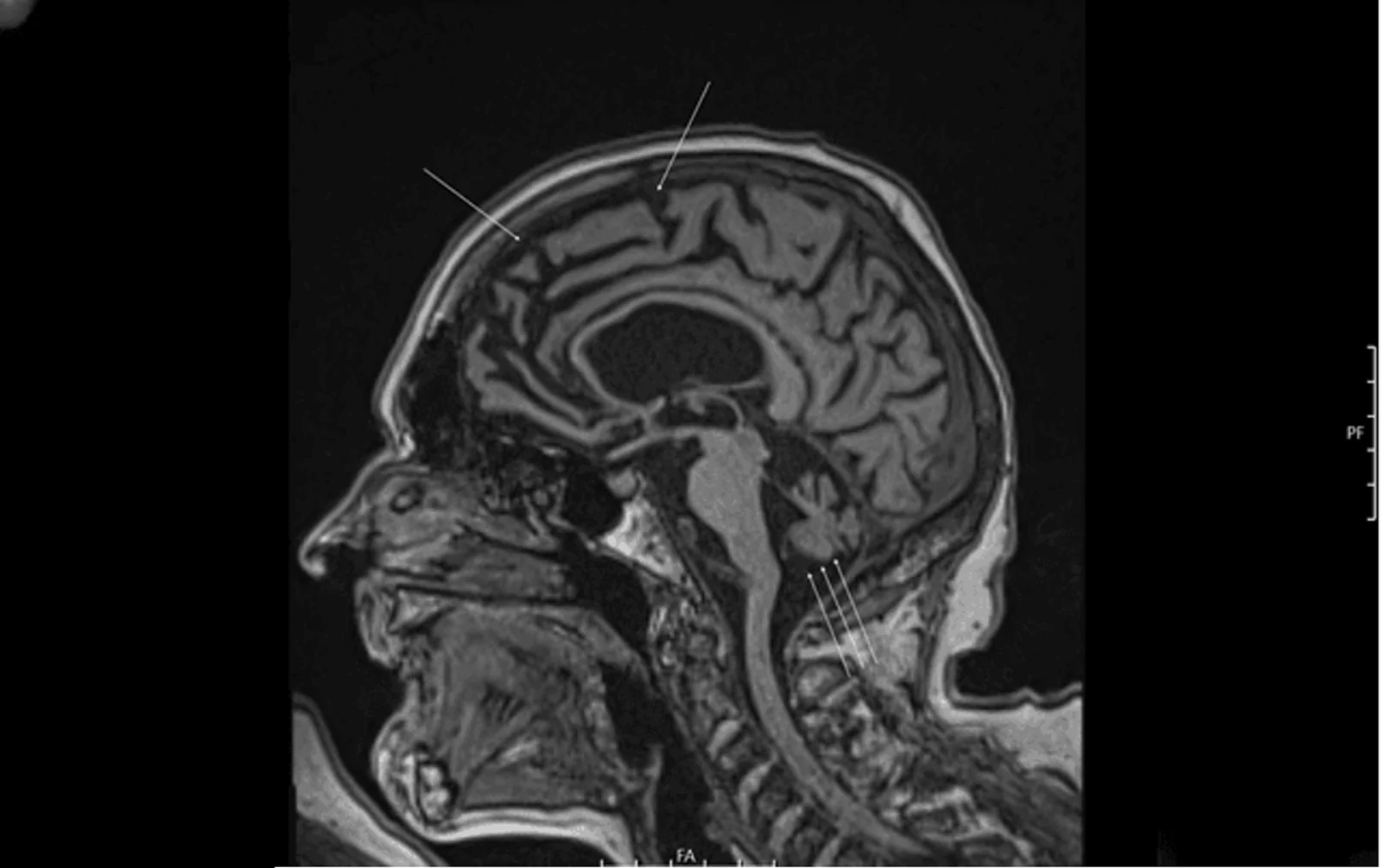
MRI of the head performed at the age of 76. White arrows show generalized brain volume loss with striking frontotemporal atrophy and diffuse cerebellar atrophy.

**Figure 3:**
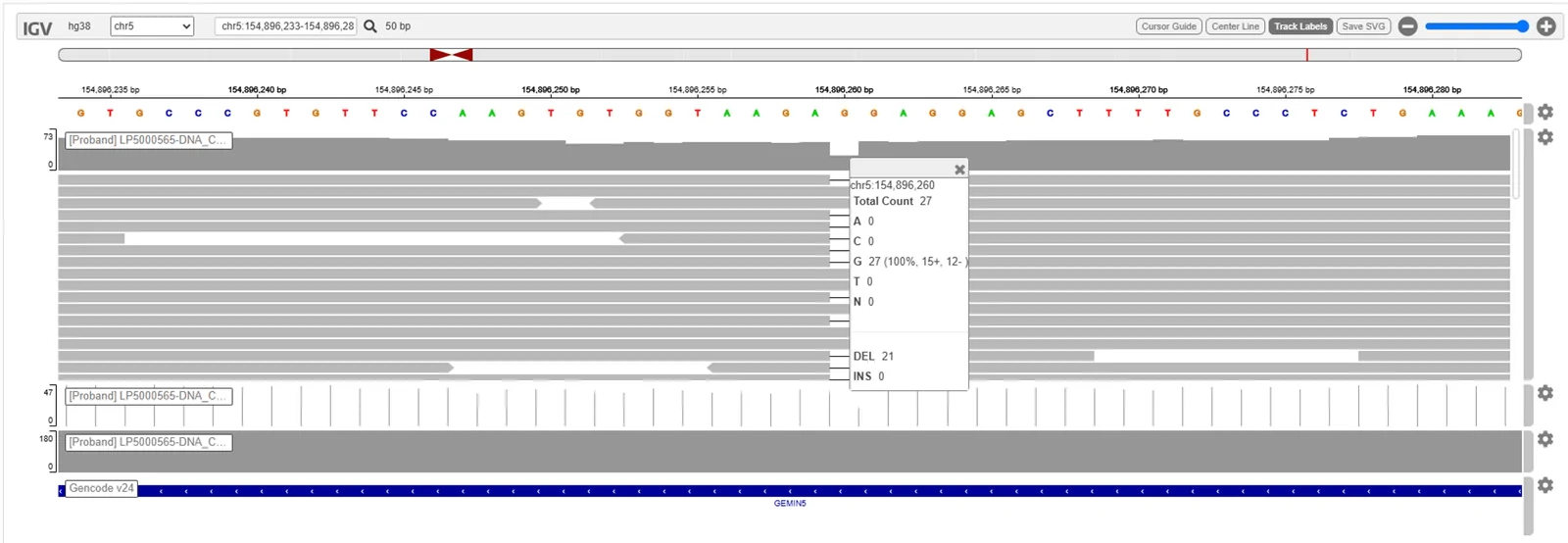
GEMIN5 pathogenic variant: NM_015465.5:c.3429del p.(Ser1144LeufsTer17) / chr5(GRCh38):g.154896259:AG:A. This figure was generated using Integrative Genomics Viewer (IGV; Broad Institute of MIT and Harvard, Cambridge, MA, USA).

**Figure 4:**
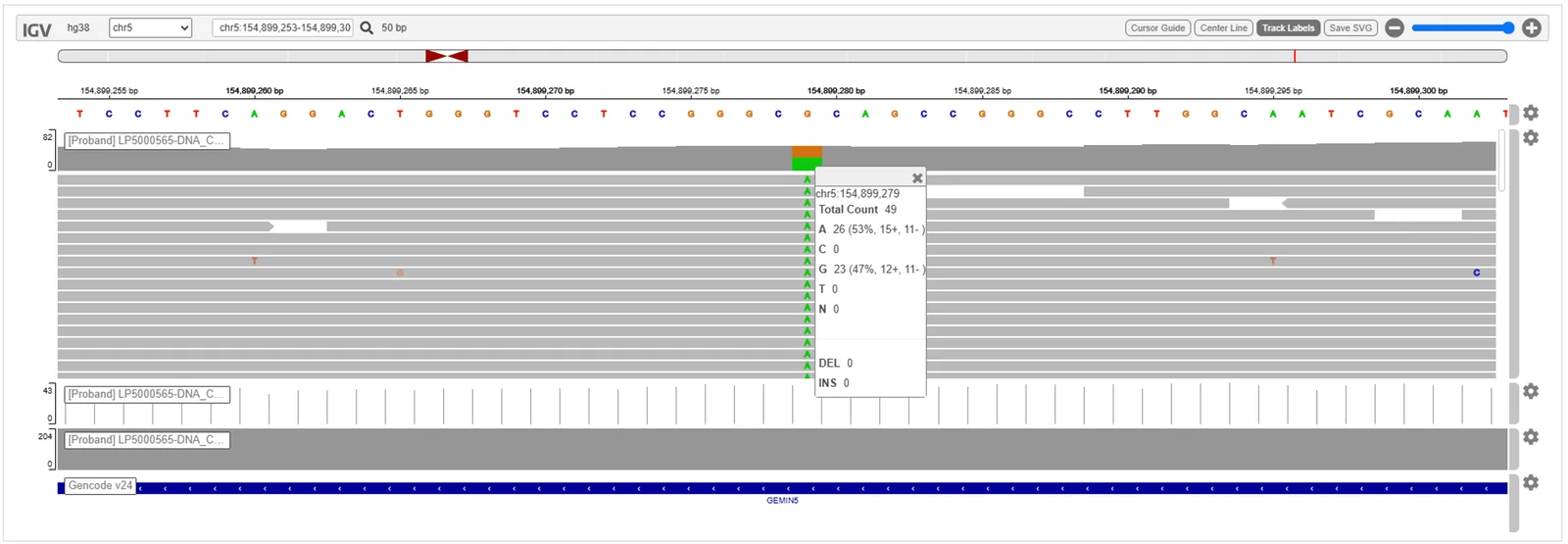
GEMIN5 gene heterozygous variant: NM_015465.5:c.3046C>T p.(Arg1016Cys) / chr5(GRCh38):g.154899279:G:A. This figure was generated using Integrative Genomics Viewer (IGV; Broad Institute of MIT and Harvard, Cambridge, MA, USA).

MRI of the head at the age of 76 showed generalized brain volume loss with progressive diffuse cerebellar atrophy and striking frontotemporal atrophy (Figure 2). Whole-genome sequencing revealed two variants in the GEMIN5 gene: a heterozygous pathogenic (class 5) loss-of-function variant, NM_015465.5(GEMIN5): c.3429del p.(Ser1144fs), and another heterozygous variant, NM_015465.5(GEMIN5): c.3046C>T (p.Arg1016Cys), which is a likely pathogenic (class 4) variant when in trans with a loss-of-function variant (Figures 3, 4). Whole-genome sequencing using the following panels: R58 adult-onset neurodegenerative disorders, R54 adult-onset hereditary ataxia, R60 adult-onset hereditary spastic paraplegia, and R62 adult-onset leukodystrophy did not reveal any other pathogenic variants in the genes tested.

There is no treatment or cure for GEMIN5 disorders; management is supportive. The patient was treated with Botox injections for contractures and referred for physiotherapy and speech and language therapy. Given the significant progression in her condition and functional decline, she was also referred to the palliative care team. A community DNACPR form and Advance Care Plan were implemented. She was later admitted to her local hospital with poor oral intake and aspiration pneumonia. Despite treatment with antibiotics, her condition deteriorated, and she died at the age of 79.

## Discussion

GEMIN5 is a multifunctional RNA-binding protein and the largest component of the SMN complex. The link between GEMIN5 loss-of-function variants and the development of a “neurodevelopmental delay and ataxia syndrome” caused by spliceosomal dysfunction was first described in 2021 [1]. The condition, characterized by early-onset developmental delay, central hypotonia, and cerebellar ataxia, is now known as NEDCAM but is often referred to as GEMIN5 syndrome. Affected individuals typically present in infancy with severe motor development impairment and cerebellar atrophy visible on brain MRI.

GEMIN5 encodes an RNA-binding protein that is crucial for the SMN complex. It has three domains: an N-terminal WD-repeat domain, which recognizes small nuclear RNAs (snRNAs); a tetratricopeptide-repeat (TPR)-like dimerization domain, which stabilizes the protein; and a noncanonical RNA-binding site (RBS1-RBS2), which regulates translation [3]. These domains allow GEMIN5 to ensure delivery of snRNAs to the SMN complex for assembly of small nuclear ribonucleoproteins (snRNPs), regulate translation and ribosome interactions, and modulate mRNA translation [4,5]. Dysregulation of GEMIN5 disrupts snRNP biogenesis and translation control, resulting in a multisystem neurological disorder.

Across all published case reports, the disorder has a recognizable core phenotype consisting of global developmental and cognitive delay, hypotonia in infancy, gait ataxia, and spasticity [1,6,7]. Patients usually present in infancy within the first year of life, but a minority have later onset or even adult presentation [6]. Additional features can include epilepsy. Epilepsy was observed more frequently in individuals with variants in the TPR-like and RBS regions, although this case series was limited and further study is needed [7]. GEMIN5 syndrome can also be associated with ocular manifestations such as cataracts, strabismus, and nystagmus [6], and behavioral abnormalities such as aggression, especially in older patients [2].

The disease course is typically static or progresses very slowly. However, a subset of patients exhibit progressive motor decline, spasticity, or intellectual deterioration [6]. The phenotype correlates with genotype: the presence of loss-of-function variants such as homozygous p.His913Arg can result in early death before three months of age, whereas some individuals with compound heterozygous missense variants retain the ability to mobilize independently in their 50s [2].

MRI typically shows cerebellar atrophy or hypoplasia, sometimes accompanied by corpus callosum dysgenesis [1,7]. Nerve conduction studies may show a neuropathic pattern with fibrillations and variable amplitudes mainly involving motor nerves [1]. EEG may show burst-suppression patterns in infants with epilepsy [7]. A limitation of this case report is that electroencephalography, nerve conduction studies, and electromyography were not performed, and electrophysiological assessment may therefore be valuable in future patients with GEMIN5-related disease to allow more complete phenotypic characterization. Genetic sequencing is central to diagnosis because it identifies the biallelic pathogenic variants in the GEMIN5 gene in affected individuals. In patients with complex or overlapping neurodegenerative phenotypes, clinicians should consider comprehensive genomic testing, including whole-genome sequencing, in collaboration with specialist genetic services.

Our case illustrates the clinical and molecular significance of compound heterozygous pathogenic variants in the GEMIN5 gene [6,8]. Our patient was found to carry two distinct variants: a pathogenic loss-of-function variant, c.3429del p.(Ser1144fs), and another variant, c.3046C>T (p.Arg1016Cys), which is classified as likely pathogenic when present in trans with a loss-of-function variant.

The p.Arg1016Cys missense variant lies within the TPR-like dimerization domain of GEMIN5, which is crucial for mediating protein-protein interactions [6]. Replacement of the highly polar arginine with a small, neutrally charged cysteine may disrupt domain conformation and impair dimerization, underscoring the pathogenic relevance of amino acids flanking position 1016. The p.Arg1016Cys variant has been associated with disease in reported cases only when present in trans with a loss-of-function variant. It supports a hypomorphic effect within an autosomal-recessive inheritance pattern [6].

Parental segregation testing could not be performed, as both parents were deceased by the time of diagnosis, so the in-trans configuration of the two variants was inferred rather than confirmed on the basis of the autosomal-recessive inheritance pattern consistently reported for GEMIN5-related disease. In this patient, the identification of both variants provides a clear genetic explanation for the phenotype and enables assessment of likely future prognosis and care needs. Establishing a genetic diagnosis also facilitates genetic counseling and carrier testing for family members who wish to pursue this.

## Conclusions

Our case report presents an outlier in the spectrum of reported GEMIN5 cases, given limited early-life symptoms and late progression causing progressive ataxia, spasticity, and bulbar dysfunction. It demonstrates that GEMIN5 syndrome can remain clinically mild for decades before progressing significantly in later life. Absence of early hypotonia or motor delay does not exclude GEMIN5-related pathology, especially in individuals with lifelong cognitive impairment. Progressive neuroimaging from cerebellar-predominant to generalized frontotemporal cerebral atrophy, together with the phenotype of ataxia and cognitive impairment, should prompt clinicians to consider rare genetic causes, including the presence of GEMIN5 variants.
